# Monitoring protected areas from space: A multi-temporal assessment using raptors as biodiversity surrogates

**DOI:** 10.1371/journal.pone.0181769

**Published:** 2017-07-24

**Authors:** Adrián Regos, Luis Tapia, Alberto Gil-Carrera, Jesús Domínguez

**Affiliations:** 1 CIBIO/InBIO, Research Center in Biodiversity and Genetic Resources, Predictive Ecology Group, Campus Agrario de Vairão, Vairão, Portugal; 2 Departamento de Zooloxía, Xenética e Antrolopoxía Fisica, Universidade de Santiago de Compostela, Campus Sur, Spain; 3 InForest Joint Research Unit (CEMFOR-CTFC), Solsona, Spain; 4 GREFA (Grupo de Rehabilitación de la Fauna Autóctona y su Hábitat), Monte del Pilar S/N, Majadahonda, Madrid, Spain; 5 EBX, Estación Biolóxica do Xurés, Vilameá, Lobios, Galicia, Spain; Universiteit Twente Faculteit Geo-Informatie Wetenschappen en Aardobservatie, NETHERLANDS

## Abstract

Monitoring protected areas (PAs) is essential for systematic evaluation of their effectiveness in terms of habitat protection, preservation and representativeness. This study illustrates how the use of species distribution models that combine remote sensing data and information about biodiversity surrogates can contribute to develop a systematic protocol for monitoring PAs. In particular, we assessed the effectiveness of the Natura 2000 (N2000) network, for conserving and preserving the representativeness of seven raptor species in a highly-dynamic landscape in northwest Spain between 2001 and 2014. We also evaluated the cost-effectiveness of the N2000 network by using the total area under protection as a proxy for conservation costs. Overall, the N2000 network was found to poorly represent the habitats of the raptor species. Despite the low representativeness, this network showed a high degree of effectiveness due to increased overall habitat availability for generalist and forest specialist species between 2001 and 2014. Nevertheless, additional protected areas should be established in the near future to increase their representativeness, and thus ensure the protection of open-habitat specialist species and their priority habitats. In addition, proactive conservation measures in natural and semi-natural ecosystems (in particular, montane heathlands) will be essential for long-term protection of Montagu’s harrier (species listed in the Annex I of the Bird Directive), and thus complying with the current European Environmental Legislation. This study sheds light on how the development and application of new protected area indices based on the combined use of freely-available satellite data and species distribution models may contribute substantially to the cost-efficiency of the PA monitoring systems, and to the ‘Fitness Check’ process of EU Nature Directives.

## Introduction

The global Aichi Biodiversity Targets of the Convention on Biological Diversity [1] aim to halt biodiversity loss by 2020. Target 11 states that ‘by 2020 at least 17% of terrestrial and inland water areas […] are conserved through effectively and equitably managed, ecologically representative and well-connected systems of protected areas’. Protected areas (PAs) are therefore a mainstay of the current conservation strategies that address the ongoing decline in biodiversity [2]. However, the ability of static PA networks to conserve biodiversity is often questioned, as rapid shifts in species’ distributions are occurring in response to changes in current environmental conditions [3,4]. Despite the increase in PAs in the last few decades, it is unlikely that the Aichi Biodiversity Targets will be met by 2020 [5]. The main means whereby the European Union (EU) is attempting to achieve these targets is the Natura 2000 network of PAs (henceforth ‘N2000’), comprising over 25,000 sites representing 18% of the area of the 27 Member States of the EU [6]. The N2000 network includes Special Protection Areas for wild birds (SPAs), designated by the Member States under the Birds Directive (2009/147/EC) to conserve the habitats of particularly threatened species and migratory species; and Special Areas of Conservation (SACs), designated for other taxa and habitats under the Habitats Directive (92/43/EEC). This network aims to ensure a ‘favourable conservation status’ for species and habitat types listed in the annexes of the aforementioned European directives.

Although systematic monitoring of PAs is essential for evaluating PA effectiveness in terms of maintaining habitat protection, preservation and representativeness [7], it is rarely carried out. In this context, satellite remote sensing is an extremely powerful tool to assist systematic monitoring processes as it enables coverage of large, remote and non-sampled areas over different time periods, thus providing a continuous source of environmental information [8]. The usefulness of remote sensing for mapping and delineation of land cover categories within and around PAs has been widely demonstrated [9]. However, the correspondence between land cover and habitat is far from straightforward [10]. This limitation can be overcome by establishing links between land cover types and species’ habitat preference. Species distribution models (also called ‘ecological niche models’ or habitat suitability models) empirically correlates field observations with environmental predictor variables, helping to predict the probability of occurrence of species for non-sampled areas [11,12]. Combining remote sensing data with ground surveys in species distribution modelling may contribute to the ecological interpretation of remote-sensing data, and provide a framework for assessing the long-term conservation effectiveness by generating information about changes in species and their habitats within and outside PAs [13,14]. Conservation funds are often limited; however, the combined use of remote sensing and species distribution modelling might be a cost-effective tool for monitoring temporal changes in biodiversity [15,16]. In addition, the increasingly important role of remote sensing in protocols for long-term monitoring of biodiversity may help to strengthen the link between satellite remote sensing and conservation biology [17–19]. Nonetheless, the number of multi-disciplinary studies integrating remotely-sensed data and habitat suitability models for monitoring the effectiveness of PAs remains very low [20,21].

In this study, we illustrate how species distribution modelling that integrates satellite remote sensing data and ground level information about biodiversity surrogates can contribute to the development of a systematic protocol for multi-temporal monitoring of PAs. In light of budget restrictions, the first step requires clear choices about the features to be used as surrogates for overall biodiversity in the assessments [7]. Raptors are ideal species for evaluating the effectiveness of PAs, as their ecological requirements usually encompass the needs of many other species [22]. We assessed the effectiveness of three types of PAs (Special Protection Areas-SPAs, Special Areas of Conservation-SACs and the entire N2000 network) for conserving (henceforth ‘**effectiveness**’) and representing (henceforth ‘**representativeness**’) seven raptors species in a highly dynamic, fire-prone landscape located in northwestern Spain between 2001 and 2014. We also evaluated the cost-effectiveness of the different types of PAs (henceforth ‘**efficiency**’), using the total area under protection as a proxy for conservation costs [23]. We expect that these three indices of conservation effectiveness will vary between the three systems of PAs. More specifically, we expect SACs to be the least effective in conserving the priority areas for target species because this system is not based on bird protection and is therefore prone to a lack of representativeness. By contrast, we hypothesize that the SPA system will be more efficient (i.e. providing a higher level of protection per unit area) in protecting the priority areas for most bird species, especially those at most risk, because these areas are specifically designed to preserve bird populations (including many raptor species). However, we also expect that SPAs and SACs will complement each other, leading to a more efficient N2000 network for protecting raptor species than the individual PA systems alone.

## Material and methods

**Fig 1:**
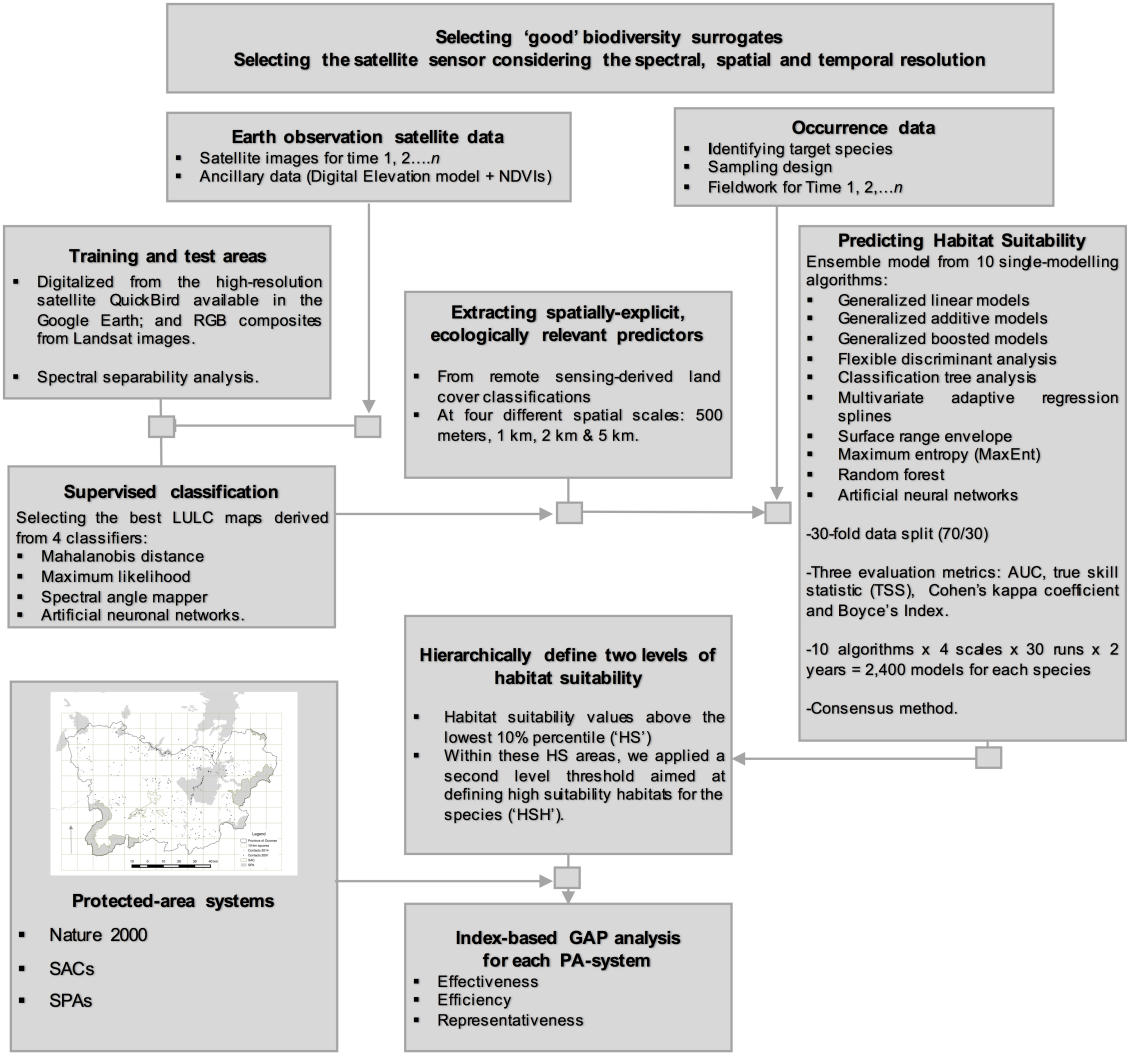
Conceptual framework of the systematic protocol for the multi-temporal monitoring of protected areas systems.

In this study we propose a framework to contribute to the development of a systematic protocol for the multi-temporal monitoring of PAs by combining remote sensing data, species distribution models and protected area indices with ground-level information about biodiversity surrogates. The modelling framework is structured in five iterative steps, as described below (sections 2.2–2.6) (see Fig 1). However, the approach is flexible because different methods can be used within each step. The framework provides verifiable, repeatable and standardized information for medium- and long-term monitoring of PAs, applicable to different periods and PA systems.

### Study region and protected-area systems

This study was conducted in Ourense province, southeast of Galicia in northwest Spain (*c*. 7,281 km^2^). The study region is located in the transition zone between the Mediterranean and Eurosiberian biogeographic zones, in the proximity of the Atlantic coast [24]. The study area is representative of the landscapes of southern Europe, which have undergone a gradual abandonment since the mid-twentieth century caused by the cessation of the traditional agropastoral activities, and the rural exodus. As consequence, the historical landscape characterized by complex field mosaics of crops and woodlands have been progressively replaced by homogeneous landscapes mainly dominated by forests (both oak forest and pine plantations) and shrublands. Agricultural areas represent less than 8% of the area (for a detailed description of vegetation composition, see section 2.4) [25]. The area is also subjected to a high frequency of human-induced fires traditionally linked to the long-standing socio-economic difficulties of rural communities (e.g., vandalism, pyromania, revenge, land use change attempts) [26], resulting in an unstable and highly dynamic system.

**Fig 2:**
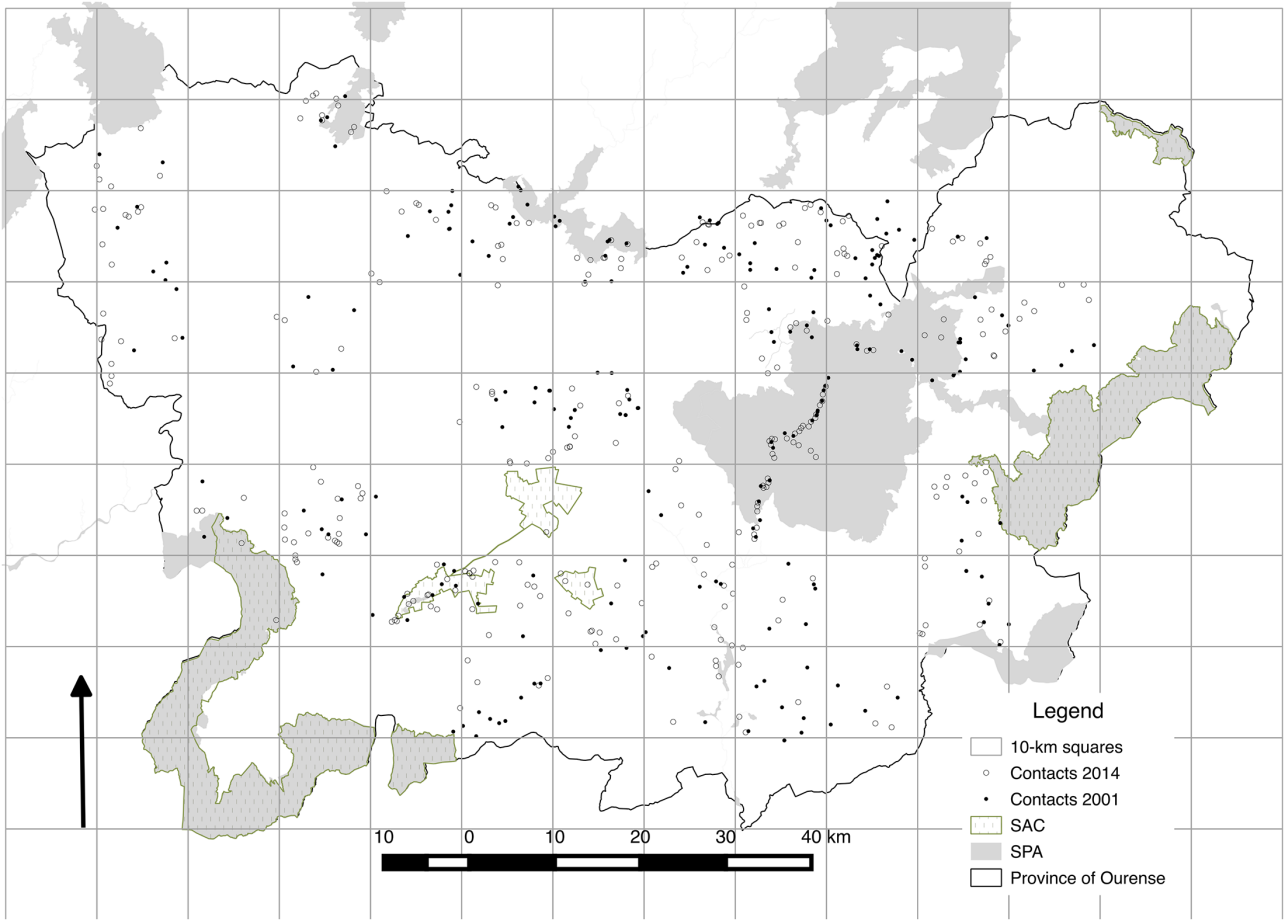
Study area, species records (both in 2001 and 2014) and protected-area systems (Natura 2000 network, SPAs and SCAs).

In the province of Ourense, 11 SACs have been designated since 1999 for the protection of habitats and species listed in the annexes of the Habitats Directive and 4 SPAs for the protection of birds included in the Birds Directive [27]. SACs and SPAs cover respectively 16.6% (*c*. 120,000 ha) and 8.3% of the study region (*c*. 60,000 ha). Their total combined extent (N2000) is 130,000 ha, and covers 17.9% of the study area (Fig 2).

### Selecting biodiversity surrogates

Biological surrogates are often used as proxies for assessing overall biodiversity when designing conservation assessment plans, saving conservation managers money and time [28–30]. In light of budget restrictions, the first step often requires clear choices about the features to be used as surrogates for overall biodiversity assessments [7]. In this sense, top predators such as raptors (recognized as flagship and umbrella species) are ideal species for evaluating the effectiveness of PAs, since their ecological requirements usually encompass the needs of many other species [22]. These species can act as valuable indicators of changes and stresses in ecosystems, as they are sensitive to changes in land-use, habitat loss and fragmentation. Conservation efforts focused on their preservation therefore usually imply an improvement in the protection of the whole region [22,31].

### Sampling design and target species

Road-based sampling surveys were carried out in the study area between May and August 2001 and 2014. For this, we divided the study area into 66 sampling units comprised of 10-km x 10-km grid cells (Fig 2). Grid cells with more than 50% of their surface outside of the province were not sampled. A final set of 34 grid cells representing all land cover types [32] was selected (c. 52% of the study area). In each grid cell, a road survey of 40 km was conducted from a 4 x 4 vehicle travelling at a speed of 30–40 km/h and covering a total of 1,360 km. In all, we conducted 140 hours of observations, always between 2 hours after sunrise and two hours before sunset. The same methods were followed in both study periods (2001 and 2014) in order to keep observations consistent and to minimize any biases associated with migratory behaviour or phenological timing. Permissions to conduct our censuses of raptors in the field were provided by “Dirección Xeral de Patrimonio Natural” da Xunta de Galicia. We did not collect or manipulate raptors in this study. We collected 327 geo-located observations of raptors in 2001 and 446 observations in 2014 for a total of 773 observations of 18 species of raptors (Fig 2). Of these 18 species, we selected those species with more than 10 presences for statistical reasons (S1 and S2 Datasets). Hence, we focused on 7 species, which exhibit different degrees of habitat specialization ranging from open habitats (i.e. shrubland and agricultural land) to forest land (Table 1; S1 Appendix).

**Table 1:** List of target species, number of observations and their European conservation status. N2001 shows the number of observations in 2001, and N2014 in 2014. Species of European Conservation Concern (SPEC) categories according to [33]: SPEC 3 (Not concentrated in Europe but with Unfavourable Conservation Status); Non-SPEC (Not concentrated in Europe and with Favourable Conservation Status). Those species included in Annex I of the Bird Directive (2009/147/EC) are indicated. ABB (abbreviation).

Species	Scientific name	ABB	N 2001	N 2014	SPEC	Annex I
**Honey buzzard**	*Pernis apivorus*	PAPI	11	18	Non-SPEC	included
**Black kite**	*Milvus migrans*	MMIG	24	31	SPEC 3	included
**Short toed eagle**	*Circaetus gallicus*	CGAL	25	20	SPEC 3	included
**Montagu´s harrier**	*Circus pygargus*	CPYG	53	29	Non-SPEC	included
**Common buzzard**	*Buteo buteo*	BBUT	134	177	Non-SPEC	Non- included
**Booted eagle**	*Hieraaetus pennatus*	HPEN	4	15	SPEC 3	included
**Common kestrel**	*Falco tinnunculus*	FTIN	42	34	SPEC 3	Non- included

### Remotely-sensed data

The land use/land cover (LULC) composition for 2000 and 2014 was derived from optical multispectral bands (30-meter resolution) of Landsat 7 Enhanced Thematic Mapper plus (ETM +) (29 March 20 2000 and 24 June 2000) and Landsat 8 Operational Land Imager (OLI) sensors (19 March 2014 and 9 July 2014). The Landsat scenes captured during spring and summer (e.g. March and July images) were considered to enhance seasonal discrepancies in the phenology of deciduous species. All Landsat scenes were processed to Standard Terrain Correction (Level 1T), which provides systematic radiometric and geometric accuracy by incorporating ground control points while employing a Digital Elevation Model (DEM) for topographic accuracy. We downloaded all scenes from the United States Geological Survey (USGS) Global Visualization Viewer (http://glovis.usgs.gov). We also included ancillary data during the classification procedure to improve the overall accuracy of the resulting maps [34]: 1) vegetation indices (NDVI; [35]) were calculated for each image to enhance the contribution of vegetation in the spectral response and mitigate other factors such as soil, topography, lighting conditions and atmosphere [36]; and 2) topography information from the Advanced Spaceborne Thermal Emission and Reflection Radiometer (ASTER) Global Digital Elevation Model (GDEM) (http://reverb.echo.nasa.gov/).

In order to produce several LULC maps, we applied a maximum likelihood classifier, the most widely-used supervised classification strategy [34], and other approaches that provided insights into the comparison of categorical maps (mahalanobis distance, spectral angle mapper and artificial neuronal networks) (see S2 Appendix for global accuracy, and confusion matrices of the LULC maps for 2000 and 2014). Eight relevant LULC types were recognised during the fieldwork: (1) deciduous forests, dominated by native oak forest (*Quercus robur* and *Q*. *pyrenaica*, which constitute the climax vegetation in the region), chestnut groves (*Castanea sativa*) and riparian forests (*Betula* spp.); (2) coniferous forests, dominated by pine plantations (*Pinus sylvestris* and *P*. *pinaster* which are a legacy of past forestry policies aimed at increasing wood production); (3) closed shrubland, i.e. shrubland and heathland covered by more than 50% of shrub species (*Cytisus* spp., *Ulex* spp. and *Erica* spp.); (4) open shrubland, i.e. rocky soil with less than 50% cover by shrub species and including areas of sparse vegetation resulting from fire events and intensive forest logging (clear-cutting); (5) meadow and fallow land; (6) arable or farming land (being or capable of being tilled for crop production); (7) water surfaces (dams and rivers); and 8) urban patches. Water surfaces and urban patches were considered constant because of very low values of change at a broad scale throughout the study period.

Training and validation areas for the supervised classification procedure were selected for each of the eight classes considered (see S2 Appendix). For each class, these were digitized by photointerpretation of high-resolution images derived from the QuickBird satellite (available in Google Earth: for more details, see https://www.digitalglobe.com/) and different RGB composites obtained by combining Landsat satellite bands. The transformed divergence index, a measure of spectral separability between classes, was used to assess the quality of statistics prior to image classification [37]. This index was computed over the training (and validation) areas to ensure maximum spectral separability (values higher than 1.99) between the different classes.

Training and validation areas based on Google Earth images were defined using Quantum GIS 2.4 software. Evaluation of the spectral separability, classification procedures, and the subsequent accuracy of the generated maps was performed using Envi 4.7 sp1 software.

### Predicting high suitability habitats

Species distribution modelling enabled empirical correlation of diurnal raptor occurrence (presence/absence) data with the remotely sensed data. Occurrence data consisted of all presence records gathered from the field surveys in 2001 and 2014. Pseudo-absence data (also known as ‘back-ground’ data), are usually drawn at random from the entire region, whereas presence data is often spatially biased toward easily accessed areas. Since the spatial bias generally results in environmental bias, the difference between presence data and background sampling may lead to inaccurate models [38]. To correct the estimation, pseudo-absences were taken from the presence points of the other species recorded during the same surveys. As the bias in the sampling design is the same for all species, better results can be obtained by using pseudo-absences within the presence points of the other species (also called 'target-group background', see [38,39]) rather than using randomly selected pseudo-absences. Species with less than 10 presences, however, were not considered for statistical reasons such as the risk of model overfitting [40,41]. LULC data covariates consisted of the percentage (%) of area occupied by each LULC class. To account for the different requirements of our raptor species in terms of home range and habitat use, we calculated the percentage (%) of area occupied by each LULC class within four levels of habitat characterization (500-m, 1-km, 2-km, 5-km radii surrounding each observation) (S1 and S2 Datasets). The radii sizes were chosen based on recent literature to ensure the inclusion of the different spatial organization levels used by raptors during the breeding season: the nest area, the post-fledging family area, and the foraging area [42,43].

Habitat suitability models were developed using the BIOMOD2 package (R-package ‘Biomod2’) [44]. All modelling algorithms available in BIOMOD2 were used: generalized linear models (GLM), generalized additive models (GAM), generalized boosted models (GBM, also known as boosted regression), flexible discriminant analysis (FDA), classification tree analysis (CTA), multivariate adaptive regression splines (MARS), surface range envelope (SRE, a.k.a. BIOCLIM), maximum entropy (MaxEnt), Breiman and Cutler’s random forest for classification and regression (RF), and artificial neural networks (ANN) [44]. The combined use of different modelling algorithms has proven a successful approach for fitting the inherent uncertainty of individual models and providing more informative and ecologically correct predictions [45]. Ensemble models were trained and evaluated by splitting data into calibration and validation subsets, including 70% and 30% of the data, respectively. We randomly repeated this procedure 30 times to yield predictions independent of the training data [46]. The predictive performance of these models was evaluated using the area under receiver operating characteristic curve (AUC) [46], true skill statistic (TSS) [47] and Cohen’s kappa coefficient [48]. The ensemble models were constructed using the weighted mean of probabilities option [45,49]. All procedures were repeated for each species, habitat characterization level and year (2400 single models for each species). The ensemble models were directly projected at 500-m grain size [50,51] so as to: (1) calculate all protected area indices at the same spatial resolution, independently of the habitat characterisation level; and (2) provide spatial projections fine enough to ensure a realistic spatial representation of the protected areas and thus reduce the risk of over-representing the original extent of PAs that could have been caused by rasterizing the PA borders at broader resolutions. Model projections were evaluated with the Boyce’s index, that only requires presences and measures how much model predictions differ from random distribution of the observed presences across the prediction gradients [52]. Boyce’s index was found to be the most appropriate metric in the case of presence-only models [53]. It is continuous and varies between -1 and +1. Positive values indicate a model which present predictions are consistent with the distribution of presences in the evaluation dataset, values close to zero mean that the model is not different from a random model, negative values indicate counter predictions, i.e. predicting poor quality areas where presences are more frequent [53]. Boyce’s index values were computed using the R package ‘ecospat’ [54], and was used to identify the best model and radius of habitat characterization for each species.

Two levels of habitat suitability were defined from the probability layers and subsequently applied to each projection: (1) suitable habitats were defined as those areas with habitat suitability values above the lowest 10% percentile (henceforth ‘SH’); and (2) within these SH areas, a second level threshold (the average of the suitable values within the SH areas) was applied with the aim of identifying high suitability habitats for the species (henceforth ‘HSH’). The 10% percentile is a widely-used threshold, as the error of omission is lower at lower percentiles (i.e. the model predicts absence in areas where the species is found) and the model is more sensitive [55]. Thus, we adopted a more conservative outlook of habitat change for both species, which is appropriate given the inherent uncertainty of modelling approaches. This method has been successfully applied in identifying priority areas for bird conservation, and selecting marine Important Bird and Biodiversity Areas (IBAs) in Spain [56–58]. HSH can be interpreted in the context of the European Birds directive as the priority areas for conservation of these species.

### Index-based analysis: Effectiveness, efficiency and representativeness

Based on core concepts of conservation planning [59], we developed three indices of the effectiveness, efficiency and representativeness of the three PA systems (i.e. the entire N2000 network, SACs and SPAs) for protecting high suitability habitats of the target species between year 2001 (t1) and 2014 (t2):

1. **Effectiveness index**, defined as the proportion of high suitability habitats (HSH) of each species (i) included within each system (s) at the end (t2) of the time interval relative to the HSH at the beginning (i.e. year 2001) (t1):
  $$Effectiveness\;\left(i-s\right)=\frac{HSH\left(t2\right)}{HSH\left(t1\right)}$$
2. **Efficiency index**, defined as the proportion of HSH of species (i) at each time interval (t) relative to the surface of each system (s) (as a proxy for conservation costs):
  $$Efficieny\;\left(i-s\right)=\frac{HSH\left(t\right)}{Surface\left(s\right)}$$
3. **Representativeness index**, defined as the proportion of HSH of each species (i) included within each system (s) relative to the HSH in the entire study area (total HSH).
  $$Representativeness\;\left(i-s\right)=\frac{HSH\left(s\right)}{Total\;HSH}$$

In addition, we calculated overall indices for the entire set of raptors, weighted by the threat status (T) of each species (i):

$$Weighted\;Effectiveness=\;\frac{\sum_{i}^{n}\left(Effectiveness\;\left(i-s\right)\right)\;x\;T}{\sum_{i}^{n}T}$$

$$Weighted\;Efficiency=\;\frac{\sum_{i}^{n}\left(Efficiency\;\left(i-s\right)\right)\;x\;T}{\sum_{i}^{n}T}$$

$$Weighted\;Representativeness=\;\frac{\sum_{i}^{n}\left(Representativeness\;\left(i-s\right)\right)\;x\;T}{\sum_{i}^{n}T}$$

where the threat status (T) was estimated by taking into account inclusion of the species in the Annex I of the Bird Directive and their European conservation status (SPEC category) (Table 1):

T=Annex  x SPEC

where the inclusion in the Annex 1 or in the SPEC category equals 2 (non-inclusion and non-SPEC equals 1).

Finally, changes in HSH for the whole study area and within the N2000 network were calculated as the difference between HSH at the end (t2) of the time interval relative to the HSH at the beginning (t1). All spatial analyses were conducted using the ‘raster’ package, and represented with ‘ggplot2’ and ‘plotrix’ packages in R [60].

## Results

### Model performance

**Fig 3:**
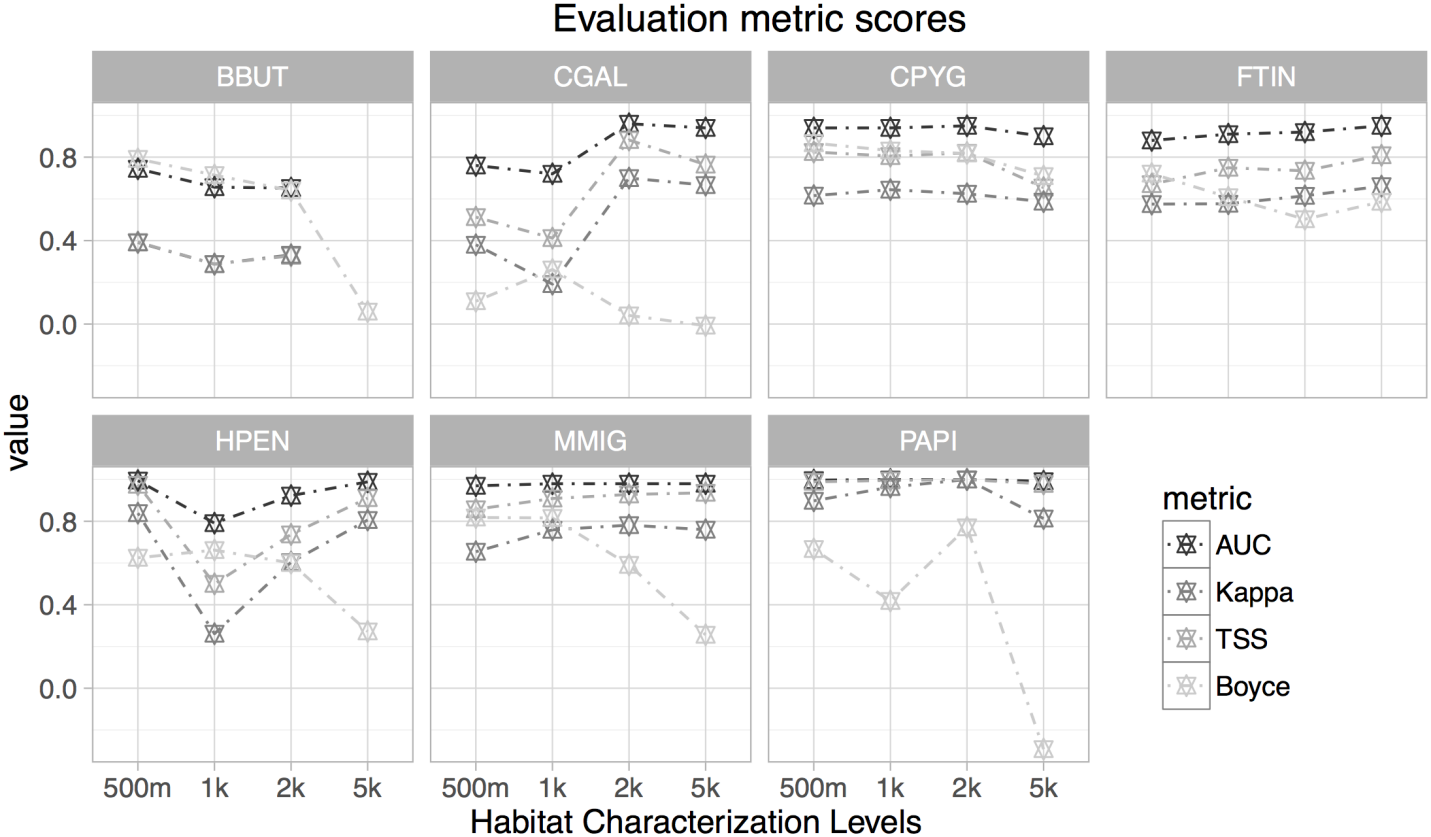
Mean evaluation metric scores for each species and habitat characterization level derived by averaging the values obtained for each period. The following three evaluation indices were calculated: area under the ROC curve (AUC), True skill statistic (TSS), Cohen’s kappa coefficient (Kappa) and Boyce’s index (Boyce). For full names of the species, see Table 1.

The ensemble habitat suitability models, based exclusively on remotely sensed data, showed a good predictive ability (AUC_MEAN_ = 0.90, AUC_SD_ = 0.10; TSS_MEAN_ = 0.75, TSS_SD_ = 0.21; Kappa_MEAN_ = 0.63, Kappa_SD_ = 0.21; and Boyce_MEAN_ = 0.70, Boyce_SD_ = 0.20, Fig 3). Only the scores for the most generalist species (Short-toed eagle *Circaetus gallicus*) indicated low accuracy (AUC < 0.75; Boyce < 0.5). The predictive capacities of the models were highly dependent on the habitat characterization level (Fig 3). Comparison across the four levels of habitat characterization identified models developed with 500-m radii as the best approaches for Montagu´s harrier *Circus pygargus*, common kestrel *Falco tinnunculus*, Black kite *Milvus migrans* and common buzzard, with 1-km radii for Booted eagle *Hieraatus pennatus* and Short-toed eagle, with 2-km radii for Honey buzzard *Pernis apivorus*. The habitat suitability models constructed by characterizing the habitat within 5-km radii showed the lowest values in terms of model performance, so they were not considered in the PA assessment (Fig 3).

### Representativeness, effectiveness and efficiency of the protected-area systems

**Fig 4:**
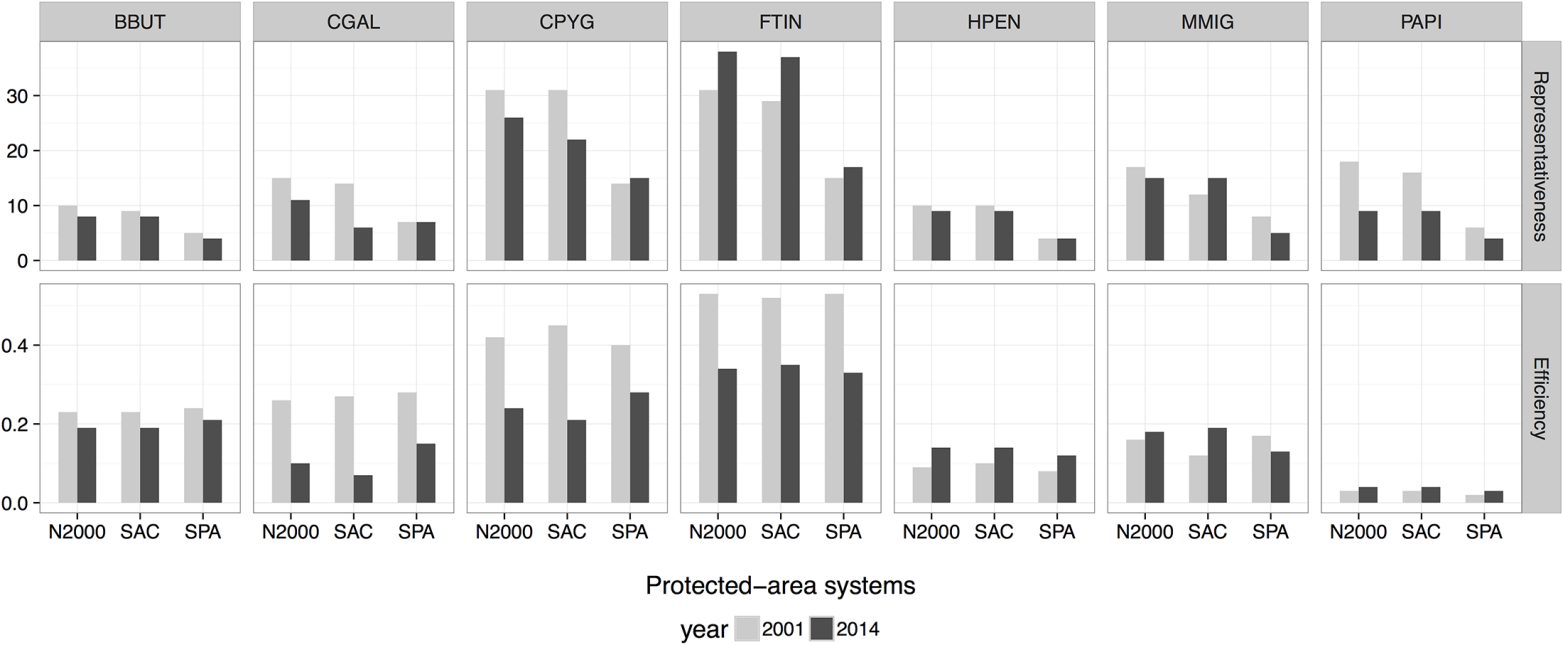
Representativeness and efficiency of each protected-area system (the whole N2000 network, SPAs and SCAs) for year 2001 and 2014. 1) Representativeness, showing the proportion (%) of high suitability habitat (HSH) for each targeted species included within each protected-area system; 2) Efficiency, defined as the percentage of HSH of each species relative to the surface of each system. For full names of the species, see Table 1.

**Fig 5:**
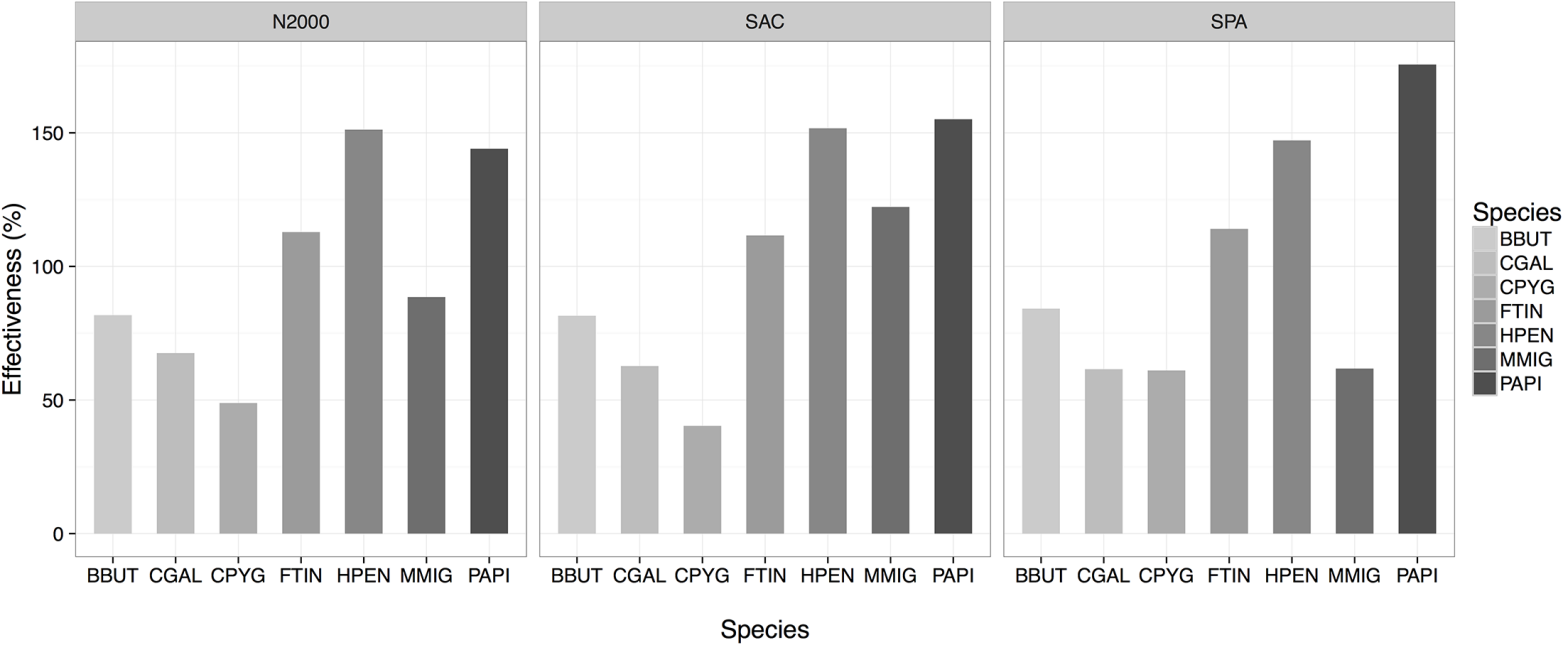
Effectiveness of each protected-area system (the whole N2000 network, SPAs and SCAs). Effectiveness, defined as percentage (%) of high suitability habitats (HSH) for each targeted species in 2014 relative to the HSH in 2000 within each PA system. For full names of the species, see Table 1.

**Fig 6:**
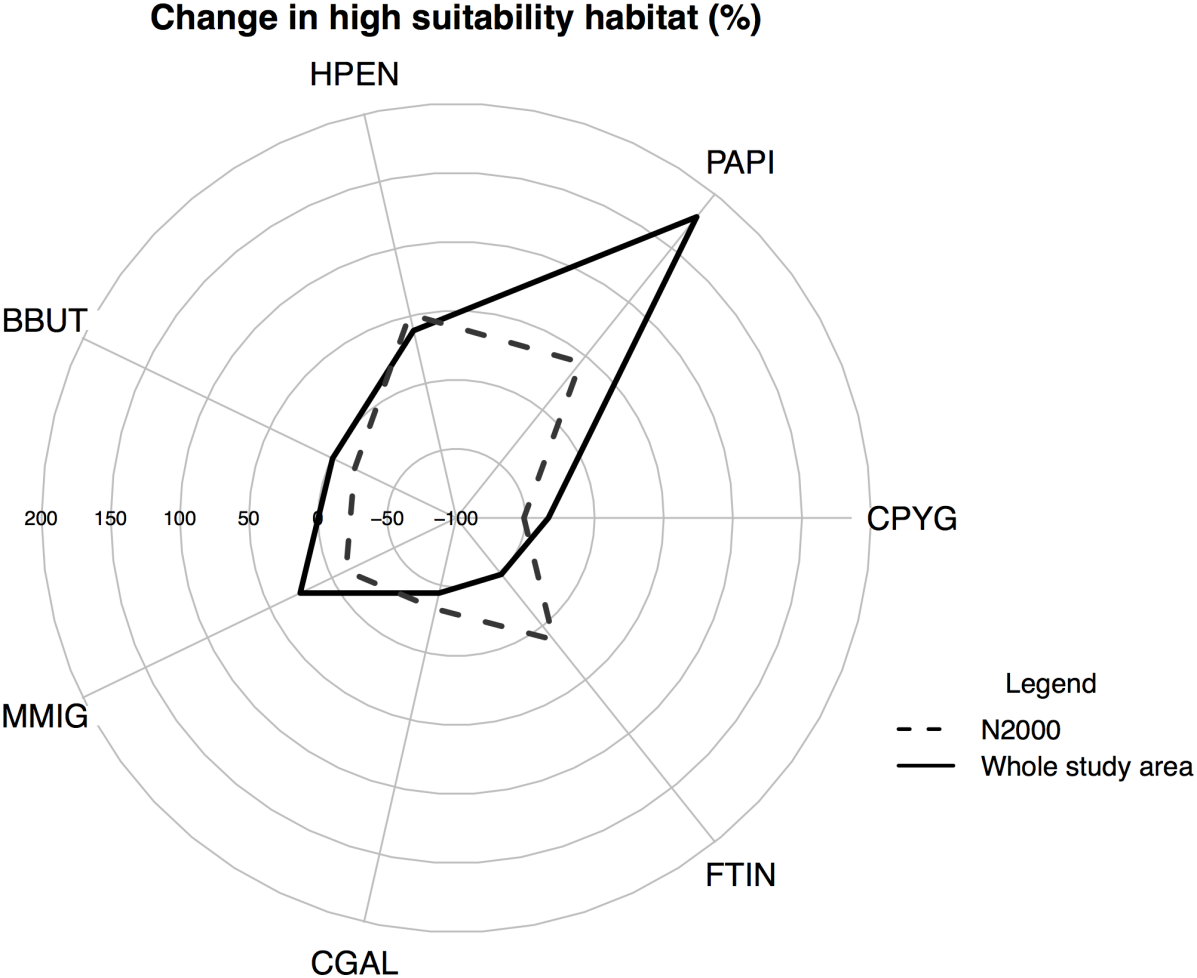
Changes (%) in the coverage of high suitability habitats for each targeted species within the Nature 2000 network (dashed line) and in the whole study area (black line) between 2001 and 2014. For full names of the species, see Table 1.

The estimated indices related to the protection of high suitability habitats of the target species showed variations among the seven species and the three systems of PAs (Figs 4 and 5). Overall, the high suitability habitats for the whole set of raptors were poorly represented in all three PA systems, especially by the SPAs (the smallest of the three protected area systems) (Table 2 and Fig 4). In particular, only two raptor species (Montagu´s harrier and Common kestrel) strongly linked to open habitats (shrubland and farmland, see S1 Appendix) were well represented (with values higher than 20%; see Fig 4). Nevertheless, these two species, together with Short-toed eagle, were the most affected by the loss of high suitability habitats in the study area (see black line in Fig 6). More importantly, the representativeness was found to decrease between year 2001 and 2014 for all species, except for Common kestrel (Fig 4).

**Table 2:** Overall representativeness, effectiveness and efficiency of the three protected-area systems. The protected-area systems are: the entire N2000 network (N2000), the Special Protection Areas (SPAs), and the Special Areas of Conservation (SACs). The index values are weighted by the threat status of each target species (see Table 1).

	Protected-area system (in %)		
Index	N2000	SCAs	SPAs
Weighted Representativeness	15	14	7
Weighted Effectiveness	95	101	95
Weighted Efficiency	16	15	20

In general, the three protected area systems were similar in terms of effectiveness and efficiency (Table 2). Overall, all three PA systems showed high effectiveness, but low efficiency, in protecting optimal habitats for raptors (Figs 4 and 5). The high effectiveness is due to an overall increase in the availability of high suitable habitats between 2001 and 2014 (Fig 6; for all species except for Montagu´s harrier, Common kestrel and Short-toed eagle). Again, the SPAs were less effective than the SCAs in protecting the priority areas for most of the species (Table 2, Fig 5). However, the SPA system was found to be the most efficient (i.e. it provided the highest level of protection per unit area) (Table 2), especially for the Common kestrel (Fig 4). The N2000 network has clearly helped to mitigate the habitat loss that this species has been suffering in the study area (Fig 6) by maintaining habitats associated with the agricultural lands (S3 Appendix). However, the efficiency of the SPAs decreased between 2001 and 2014 (Fig 4), which can compromise its future role in protecting the most threatened species (i.e. Common kestrel and Montagu´s harrier).

## Discussion

This study proposes a framework that will contribute to developing a cost-effective and systematic protocol for monitoring PA networks. We showed how satellite remote sensing can be combined with ground-level biodiversity data, species distribution models and protected area indices to enable verifiable, repeatable and standardized information for medium- and long-term monitoring, applicable to different time periods and protected-area systems (Fig 1). Spatial conservation priorities are highly sensitive to choice of biodiversity surrogates [28,61]. In this sense, previous research provides evidences of a tight association, at least in some biological systems, between top predators and high biodiversity, especially if the species differ widely in their diet and habitat associations which can offer much complementary in terms of species composition [62]. Our target raptor species were found to be associated with different habitat types, from open habitats (including farmlands and shrublands) to forests. Our raptor species also showed different degree of specialization, from specialist species such as Montagu´s harrier (tightly associated with closed shrubland) or Common kestrel (more closely correlated with meadows and arable land) to more generalist ones such as Common buzzard (see S1 Appendix). In general, habitat specialists with narrow niche breadths often show a low degree of tolerance to ecosystem alteration, so they can act as a good indicators of environmental change [63]. On the other hand, top predators are also considered indicators because they are at the top of food webs and need wide home-range areas to function. Predators play an important role in ecosystems because they can determine the community structure patterns of their prey [64]. In addition, raptors have been used as ‘umbrella’ species in world conservation strategies because their protection may facilitate the conservation of great portions of unaltered habitats [65]. In fact, previous research showed that sites selected on the basis of predators held greater densities of individual birds and butterflies than other sites [65]. Focusing on top predators was also found to allow a more efficient selection of sites required to achieve a given level of species representation in PA systems [22,65]. Nevertheless, quantitative tests of the surrogate-efficacy of these indicators have been astonishingly few, and context-dependent. Thus, other authors appeals to conservation biologists to use top predators more cautiously as surrogates or indicators [66,67]. In this regard, our monitoring PA framework is sufficiently flexible to support initial multi-taxon datasets, which would improve the surrogacy effectiveness [68].

Gap analysis is commonly used in the assessment of PA systems to determine the degree to which conservation targets are achieved [7,69,70]. Here we performed a comparative gap analysis of three PA systems (N2000, SPAs and SACs) designated according to different criteria and conservation targets. This implied the use of different indices to measure their ability to fulfil protection and representation goals. To our knowledge, very few studies have evaluated the efficiency and effectiveness of PA systems at two different times after implementation [71]. From our viewpoint, a multi-temporal perspective is needed to estimate the effectiveness of protection afforded, especially under changing environmental conditions, and new protected area indices should account for this multi-temporal dimension. Otherwise, this type of evaluation would be limited to estimating the degree to which habitats and species are represented within a particular system at a certain time. Although the conservation cost per unit area is not homogeneous across space, but varies considerably among different protected sites [23], the total area protected in a given region is widely recognized as an adequate proxy for estimating cost-effectiveness [72].

**Fig 7:**
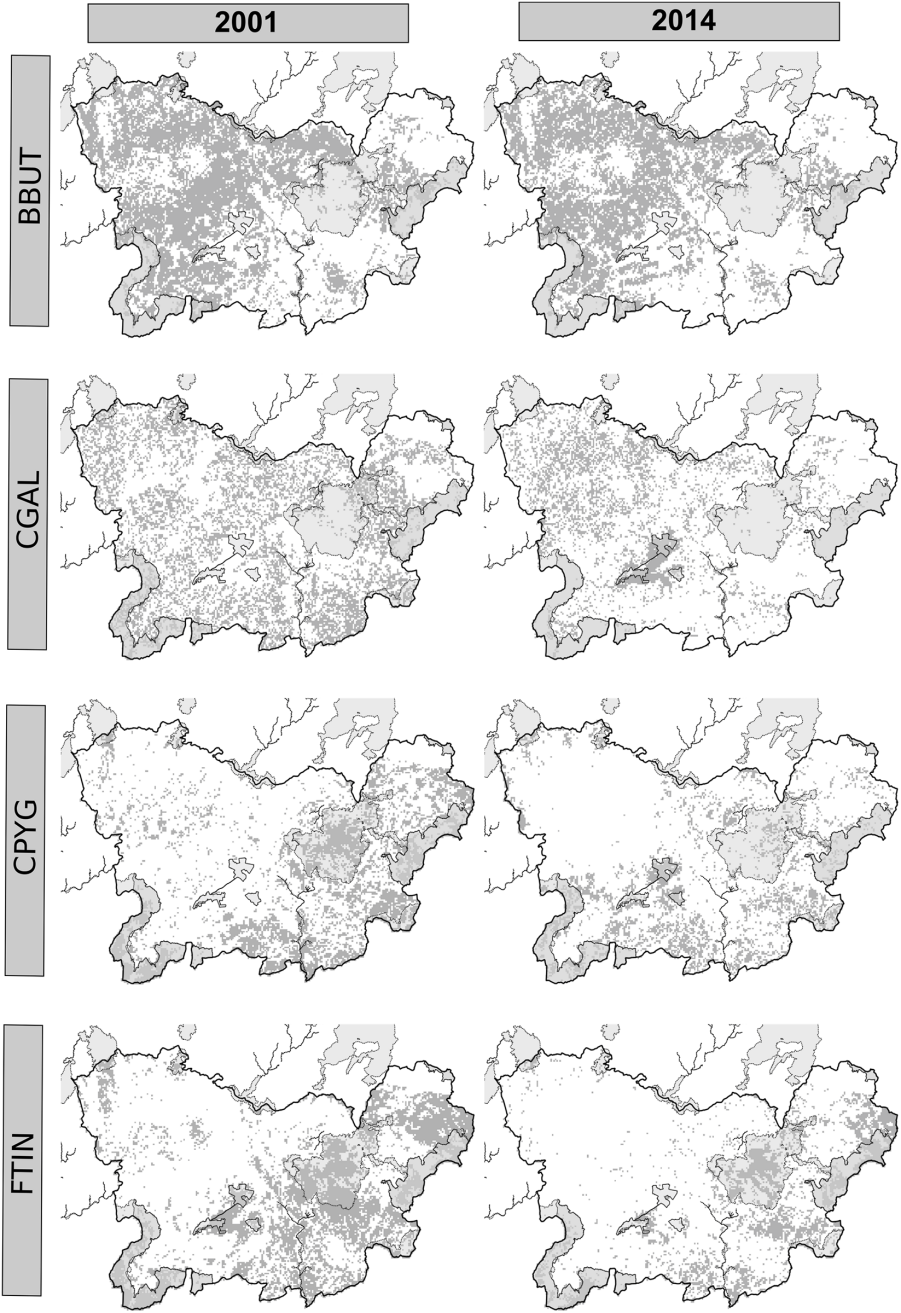
Distribution of high suitability habitats within the Nature 2000 network (grey shaded areas) in the whole study area (black line) between 2001 and 2014. Acronyms: *Buteo buteo* (BBUT), *Circaetus gallicus* (CGAL), Circus pygargus (CPYG), and *Falco tinnunculus* (FTIN).

**Fig 8:**
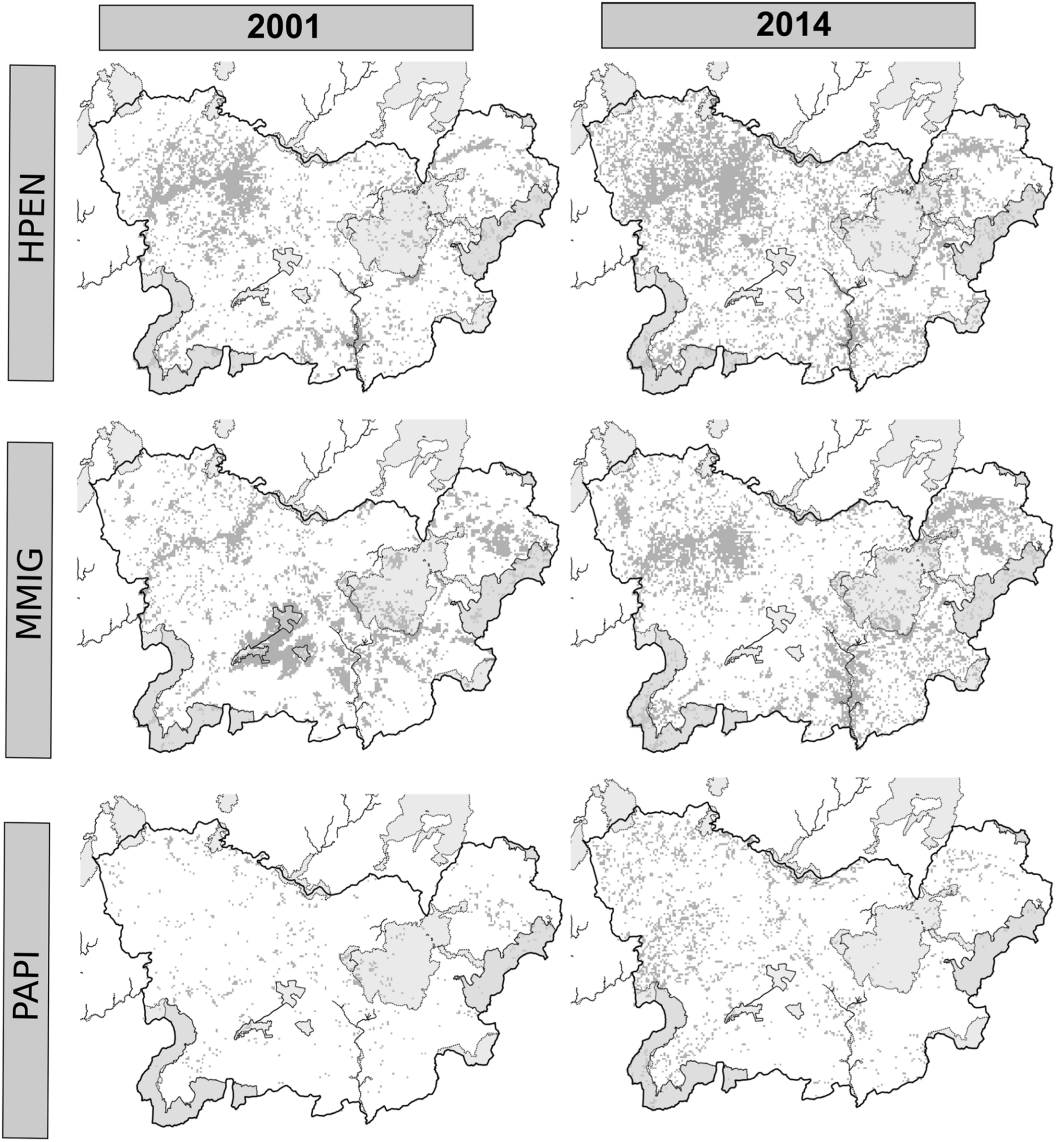
Distribution of high suitability habitats within the Nature 2000 network (grey shaded areas) in the whole study area (black line) between 2001 and 2014. [continuation]. Acronyms: *Hieraaetus pennatus* (HPEN), *Milvus migrans* (MMIG), and Pernis apivorus (PAPI).

The three PA systems showed different degrees of effectiveness and efficiency for protecting and representing the target species’ habitats. Overall, the Natura 2000 network poorly represented the habitats of the target raptor species (Table 2, Figs 4, 7 and 8). Recent studies have predicted that PA networks will remain important for future bird conservation under climate [4,73] and land cover change [58,74,75]. However, a low degree of overlap between the distribution of protected areas and threatened species could compromise the present and future role of such networks in tackling global biodiversity loss [76,77]. In the present study, the SPAs, although specifically implemented to preserve bird populations (including many raptor species) [78–80], yielded the lowest representativeness values of the three systems in the study region (Table 2). Despite the low representativeness of the Natura 2000 network, this system proved to be highly effective for protecting raptor species and their habitats (Fig 5) as a result of the overall increase in the habitat availability for most of the species between 2001 and 2014 (Figs 6, 7 and 8). These results also suggest that SCAs and SPAs complement each other to a certain extent, despite the high degree of overlap. However, efficiency values of <25% indicated that conservation of these habitats is likely very costly as more than 4 hectares are required to maintain 1 hectare of habitat (Table 2). The effectiveness, efficiency and representativeness varied greatly between the different raptor species. The least protected species (by far) was the Montagu´s harrier, which suffered a decrease in suitable habitat of more than 50% between 2001 and 2014 inside Natura 2000 network (Figs 5 and 6). By contrast, the Natura 2000 network was very effective in protecting and representing the Common kestrel’s habitat (Figs 4, 7 and 8), and only 1.8 hectares were required to protect 1 hectare of habitat (efficiency value for year 2000 of 0.53). However, the efficiency was found to decrease between year 2001 and 2014 (Fig 4), which could strongly compromise the future role of the Natura 2000 network in protecting the most threatened species (Common kestrel and Montagu´s harrier). In this regard, additional protected areas should be established in the near future to increase their representativeness, and thus ensure the protection of open-habitat specialist raptor species and their priority habitats. In addition, proactive conservation measures of natural and semi-natural ecosystems (montane heathlands) will be essential for long-term protection of Montagu’s harrier (species listed in the Annex I of the Bird Directive) [81], and thus complying with the current European Environmental Legislation and the global Aichi Biodiversity Targets of the Convention on Biological Diversity.

From a more methodological viewpoint, our modelling framework helps to overcome some limitations related to the initial biodiversity data (e.g. insufficient data, sample selection bias or presence-only data; [38]) and the modelling techniques (e.g. uncertainty associated with the algorithm selection, the consensus procedure or the model evaluation method) [49,82], while simultaneously contributing to the cost-efficiency of the monitoring system [83]. In general, resources for biodiversity monitoring are too limited to gather large sets of data including both presences and absences, so species distribution models often rely on presence-only data [84]. These modelling approaches based on presence-only data are prone to suffer from sample selection bias [85]. In this regard, developers of some of the most widely-used presence-only software (e.g. MAXENT) have begun to explore methodologies to account for sample selection bias when additional information on sampling effort is available [38,86]. Thus, the ‘target-group background’ method (which consists of treating points where other species in the same data set were recorded as background points) was found to improve average performance for several modelling techniques in comparison with classical methods based on a random selection of pseudo-absence data from the entire region [38,85]. This improvement was found to be greatest when there is strong bias in the target-group presence records [38]. More importantly, if this target group is appropriate, then users could simply use presence–absence methods rather than presence-only modelling [38,85]. Moreover, the model evaluation metric can yield inaccurate and inappropriate conclusions in many cases, especially in presence-only based models wherein pseudo-absence can contribute with an additional level of model uncertainty. Thus, true skill statistic (TSS) was found to compensate for the shortcomings of kappa statistic (inherently dependent on prevalence) while keeping all of its advantages [47]. However, TSS index is sensitive to the threshold applied to transform raw probabilities into presence-absence predictions. An alternative method for assessing the accuracy of SDMs is the area under the ROC curve (AUC)—often used as a single threshold-independent measure for model performance [46]. Despite these advantages, some authors also found AUC inappropriate because the two error components (false-presence and false-absence errors) are weighted equally [87], which can be particularly worrying when modelling with pseudo-absences [88]. In this regard, Boyce’s index is one of the most adequate metrics when dealing with presence-only data [53], as it only requires presences, is threshold-independent and offers further insights into the model quality: robustness, habitat suitability resolution and deviation from randomness [53]. In our particular case, we found that for some species (e.g. Montagu´s harrier and Common kestrel) Boyce’s index values were consistent with values from AUC, TSS and Kappa indices (Fig 3), which is in line with previous works [53]. However, for other species Boyce’s index showed very low values in comparison with the more classical ones (see e.g. Honey buzzard or Short-toed eagle at 5-km level), so this metric was decisive to select the best habitat characterization level, and modelling approach. Overall, the highly accurate scores of evaluation metrics yielded by the habitat predictions indicate the potential usefulness of remote sensing-derived land cover classifications to provide spatially-explicit, ecologically relevant predictors [8], that fully capture the habitat characteristics of most of the target species (Fig 2). This facilitates the correspondence between land cover and species’ habitat, thus overcoming one of the main limitations associated with the protected-area assessments based exclusively on remote-sensing data [10]. Overall, the best models were built by characterizing the habitat within 500-meter and 1- km radii around each observation, which is more associated with nesting areas, and the post-fledging family areas than foraging habitats [42,43]. Therefore, comparison between modelling approaches based on different levels of habitat characterization enabled identification of the best options for each species in terms of home range and habitat use, reinforcing the relevance of multiple spatial scales in species distribution modelling [89] and, therefore, in protected-area monitoring.

## Conclusions

The study confirms that the development and application of new protected area indices based on the combined use of freely-available satellite data and species distribution models can substantially contribute to the cost-efficiency of the PA monitoring systems. It also shows the relevance of considering multi-temporal, multi-level and multi-species approaches for a more comprehensive evaluation of the effectiveness, efficiency and representativeness of the protected-area networks. At regional level, the N2000 network was found to poorly represent the habitats of the raptor species. However, this network showed a high degree of effectiveness due to increased overall habitat availability for generalist and forest specialist species between 2001 and 2014. Nevertheless, additional protected areas should be established in the near future to increase their representativeness, and thus ensure the protection of open-habitat specialist raptor species and their priority habitats. In addition, proactive conservation measures in natural and semi-natural ecosystems (montane heathlands) will be essential for long-term protection of Montagu’s harrier (species listed in the Annex I of the Bird Directive), and thus complying with the current European Environmental Legislation and, in turn, with the global Aichi Biodiversity Targets of the Convention on Biological Diversity. In 2014 the European Union initiated a process called ‘Fitness Check’ of EU Nature Legislation. This process is a comprehensive and evidence-based policy evaluation aimed at assessing the effectiveness, efficiency, coherence, relevance and EU added value of the Birds and Habitats Directives in contributing to the EU Biodiversity Strategy. As the Natura 2000 network constitutes the backbone of biodiversity conservation in Europe, the application of the proposed framework at large spatiotemporal scales may contribute significantly to the assessment process. Finally, the use of this framework may also help to strengthen the link between remote sensing, ecological modelling and conservation biology.

## Supporting information

S1 Dataset Raptor presence/absence data for 2001, and percentage of each land cover type at the radii of 500 meters, 1 km, 2 km and 5 km (in CSV format). Acronyms: *Pernis apivorus* (PAPI), *Milvus migrans* (MMIG), *Circaetus gallicus* (CGAL), *Circus pygargus* (CPYG), *Buteo buteo* (BBUT), *Hieraaetus pennatus* (HPEN), *Falco tinnunculus* (FTIN), Open shrubland (OShr), deciduous forest (DeFo), meadows and fallow lands (Med), arable and farming lands (ArL), coniferous forest (CoFo), closed shrubland (CShr).
(CSV)

S2 Dataset Raptor presence/absence data for 2014, and percentage of each land cover type at the radii of 500 meters, 1 km, 2 km and 5 km (in CSV format). Acronyms: *Pernis apivorus* (PAPI), *Milvus migrans* (MMIG), *Circaetus gallicus* (CGAL), *Circus pygargus* (CPYG), *Buteo buteo* (BBUT), *Hieraaetus pennatus* (HPEN), *Falco tinnunculus* (FTIN), Open shrubland (OShr), deciduous forest (DeFo), meadows and fallow lands (Med), arable and farming lands (ArL), coniferous forest (CoFo), closed shrubland (CShr).
(CSV)

S1 Appendix Habitat preference and specialization of the raptor species. (DOCX)

S2 Appendix Global accuracy of the remotely-sensed data-derived LULC maps for 2000 and 2014 from four classification methods. (DOCX)

S3 Appendix Land use and cover change within the three protected-areas systems. (DOCX)
