# Supplementary material for: Monitoring protected areas from space: A multi-temporal assessment using raptors as biodiversity surrogates
Source: PLoS One. 2017 Jul 24;12(7):e0181769. doi: 10.1371/journal.pone.0181769 (PMC5524325; doi:10.1371/journal.pone.0181769)
Supplement: S1 Appendix — (DOCX) [file pone.0181769.s003.docx]

**Monitoring protected areas from space:** **a** **multi-temporal assessment using raptors as biodiversity surrogates**

Adrián Regos^1,2,3*^, Luis Tapia^2^, Alberto Gil-Carrera^4,5^, Jesús Domínguez^2^

**Appendix S1. Habitat preference and specialization of the raptor species.**

This supplementary material shows the correlation between raptor occurrence at broad scale (at the 10-km sampling unit level) determined in the field surveys of 2001 and 2014 with the LULC variables derived from remote sensing data (2000 and 2014). To do so, we performed a co-inertia analysis, which is a robust approach for studying species–environment relationships, especially if the variables are correlated [1]. It is therefore particularly well adapted to the study of the modifications in species–environment relationships during the 2 years of surveys [2,3]. The raptor data set comprised the occurrence (presence/absence) of seven species in 68 squares (34 plots x 2 years), and the LULC data set consisted of the percentage (%) of area occupied by each LULC category, for each of the 68 squares.

**Figure S1.** Coinertia analysis showing the habitat preference and specialization for each raptor species according to the correlation with land cover types. Acronyms: open shrubland (Oshr), deciduous forest (DeFo), meadows and fallow lands (Med), arable and farming lands (ArL), coniferous forest (CoFo), closed shrubland (CShr).

The degree of habitat preference and specialization for each raptor species was identified in the co-inertia analysis according to the correlation with land cover types (S1 Fig.): 1) the specialist species, Montagu´s harrier (CPYG; strongly associated with closed shrubland), Common kestrel (FTIN; more closely correlated with meadows and arable land), and Black kite (MMIG) and European honey buzzard (PAPI; associated with forestland); and 2) the more generalist species (not closely associated with any particular land cover type) the Common buzzard (BBUT), Short-toed eagle (CGAL) and Booted eagle (HPEN).

**References**

1. Dolédec S, Chessel D. Co-inertia analysis: an alternative method for studying species-environment relationships. Freshw Biol. 1994;31: 277–295.

2. Sirami C, Brotons L, Martin J-L. Vegetation and songbird response to land abandonment: from landscape to census plot. Divers Distrib. 2007;13: 42–45. doi:10.1111/j.1472-4642.2006.00297.x

3. Regos A, Domínguez J, Gil-Tena A, Brotons L, Ninyerola M, Pons X. Rural abandoned landscapes and bird assemblages: winners and losers in the rewilding of a marginal mountain area (NW Spain). Reg Environ Chang. Springer Berlin Heidelberg; 2016;16: 199–211. doi:10.1007/s10113-014-0740-7
