# Supplementary material for: Monitoring protected areas from space: A multi-temporal assessment using raptors as biodiversity surrogates
Source: PLoS One. 2017 Jul 24;12(7):e0181769. doi: 10.1371/journal.pone.0181769 (PMC5524325; doi:10.1371/journal.pone.0181769)
Supplement: S2 Appendix — (DOCX) [file pone.0181769.s004.docx]

**Monitoring protected areas from space:** **a** **multi-temporal assessment using raptors as biodiversity surrogates**

Adrián Regos^1,2,3*^, Luis Tapia^2^, Alberto Gil-Carrera^4,5^, Jesús Domínguez^2^

**Appendix S2. Global accuracy of the remotely-sensed data-derived LULC maps for 2000 and 2014 from four classification methods.**

This supplementary material shows the number of training and validation areas per land cover class and year (S2.1. Table), the accuracy of the Landsat data-derived maps (S2.2 Table), and the confusion matrices for 2000 and 2014 (S2.3 and S2.4 Tables). Five classification methods were compared, and the procedure yielding the best results was selected. The satellite data-derived maps for 2000 and 2014 were generated, with an overall accuracy of 97.70 and 94.89 % (Kappa coefficients of 0.97 and 0.93), using the maximum likelihood algorithm, which was the most consistent for both dates (see comparison between algorithms in S2.2 Table). Confusion matrices based on the maximum likelihood algorithm-derived maps were used to assess the classification accuracy of each year (S2.3 and S2.4 Tables).

**S2.1 Table.** Total number of training and validation areas per land cover class and year.

|  | **Training areas** | | **Validation areas** | |
| --- | --- | --- | --- | --- |
|  | **2000** | **2014** | **2000** | **2014** |
| **Water** | 961 | 963 | 955 | 1040 |
| **Open shrubland** | 481 | 1025 | 488 | 823 |
| **Deciduous forest** | 145 | 146 | 173 | 207 |
| **Meadows and fallow lands** | 402 | 659 | 390 | 652 |
| **Arable and farming lands** | 219 | 446 | 230 | 395 |
| **Coniferous forest** | 221 | 233 | 221 | 231 |
| **Closed shrubland** | 244 | 240 | 216 | 354 |

**S2.2 Table**. Global accuracy of the four algorithms applied for the Landsat images classification procedure, grouped by year and algorithm: overall accuracy and Kappa coefficient.

|  | Mahalanobis distance | Maximum likelihood | Spectral angle mapper | Artificial neuronal networks |
| --- | --- | --- | --- | --- |
| 2000 | 87.00 (0.84) | **97.70 (0.97)** | 77.95 (0.74) | 93.35 (0.91) |
| 2014 | 89.03 (0.86) | **94.89 (0.93)** | 79.66 (0.75) | 92.17 (0.90) |

**S2.3 Table.** Confusion matrices and statistical accuracy assessment defined for each land cover class in 2000. Both classification results (rows) and ground truth (columns), in percentage values.

| 2000 | Water | Open shrubland | Deciduous forest | Meadows and fallow lands | Arable and farming lands | Coniferous forest | Closed shrubland | Commission’s  errors | Omission’s  errors | Producer Accuracy | User  Accuracy |
| --- | --- | --- | --- | --- | --- | --- | --- | --- | --- | --- | --- |
| Water | **99.38** | 0.00 | 0.00 | 0.00 | 0.00 | 0.00 | 0.00 | **0.00** | **0.62** | **99.38** | **100.00** |
| Open shrubland | 0.62 | **100.00** | 0.00 | 0.00 | 0.46 | 0.00 | 0.00 | **1.43** | **0.00** | **100.00** | **98.57** |
| Deciduous forest | 0.00 | 0.00 | **100.00** | 0.00 | 0.00 | 0.00 | 11.48 | **16.18** | **0.00** | **100.00** | **83.82** |
| Meadows and fallow lands | 0.00 | 0.00 | 0.00 | **97.01** | 0.00 | 0.00 | 0.00 | **0.00** | **2.99** | **97.01** | **100.00** |
| Arable and farming lands | 0.00 | 0.00 | 0.00 | 2.99 | **99.54** | 0.00 | 0.00 | **5.22** | **0.46** | **99.54** | **94.78** |
| Coniferous forest | 0.00 | 0.00 | 0.00 | 0.00 | 0.00 | **100.00** | 0.00 | **0.00** | **0.00** | **100.00** | **100.00** |
| Closed shrubland | 0.00 | 0.00 | 0.00 | 0.00 | 0.00 | 0.00 | **88.52** | **0.00** | **11.48** | **88.52** | **100.00** |

**S2.4 Table.** Confusion matrices and statistical accuracy assessment defined for each land cover class in 2014. Both classification results (rows) and ground truth (columns), in percentage values.

| 2014 | Water | Open shrubland | Deciduous forest | Meadows and fallow lands | Arable and farming lands | Coniferous forest | Closed shrubland | Commission’s  errors | Omission’s  errors | Producer Accuracy | User Accuracy |
| --- | --- | --- | --- | --- | --- | --- | --- | --- | --- | --- | --- |
| Water | **98.96** | 0.00 | 0.00 | 0.00 | 0.00 | 0.00 | 0.00 | **0.00** | **1.04** | **98.96** | **100.00** |
| Open shrubland | 0.42 | **99.80** | 0.00 | 0.00 | 0.00 | 0.00 | 16.67 | **4.12** | **0.20** | **99.80** | **95.88** |
| Deciduous forest | 0.00 | 0.00 | **94.52** | 0.00 | 0.00 | 0.00 | 1.25 | **2.13** | **5.48** | **94.52** | **97.87** |
| Meadows and fallow lands | 0.00 | 0.00 | 1.37 | **100.00** | 0.00 | 0.00 | 0.00 | **0.30** | **0.00** | **100.00** | **99.70** |
| Arable and farming lands | 0.62 | 0.00 | 0.00 | 0.00 | **100.00** | 0.00 | 4.58 | **3.67** | **0.00** | **100.00** | **96.33** |
| Coniferous forest | 0.00 | 0.00 | 4.11 | 0.00 | 0.00 | **100.00** | 0.00 | **2.62** | **0.00** | **100.00** | **97.38** |
| Closed shrubland | 0.00 | 0.20 | 0.00 | 0.00 | 0.00 | 0.00 | **77.50** | **1.06** | **22.50** | **77.50** | **98.94** |
