# Supplementary material for: Monitoring protected areas from space: A multi-temporal assessment using raptors as biodiversity surrogates
Source: PLoS One. 2017 Jul 24;12(7):e0181769. doi: 10.1371/journal.pone.0181769 (PMC5524325; doi:10.1371/journal.pone.0181769)
Supplement: S3 Appendix — (DOCX) [file pone.0181769.s005.docx]

**Monitoring protected areas from space:** **a** **multi-temporal assessment using raptors as biodiversity surrogates**

Adrián Regos^1,2,3*^, Luis Tapia^2^, Alberto Gil-Carrera^4,5^, Jesús Domínguez^2^

**Appendix S3. Land use and cover change within the three protected-area systems.**

This supplementary material shows the main land use and cover change in the study area between 2000 and 2014 within the three protected-area systems (S3.1 Fig).

**S3.1 Fig.** Change (in hectares) between 2000 and 2014 of each land cover class inside each protected-area system: the whole Natura 2000 network (N2000), Special Areas for Conservation (SCAs) and Special Protection Areas for Birds (SPAs).
